# mARC Treatment of Hypopharynx Carcinoma with Flat and Flattening-Filter-Free Beam Energies – A Planning Study

**DOI:** 10.1371/journal.pone.0164616

**Published:** 2016-10-14

**Authors:** Katharina Bell, Jochen Fleckenstein, Frank Nuesken, Norbert Licht, Christian Rübe, Yvonne Dzierma

**Affiliations:** Department of Radiotherapy and Radiation Oncology, Saarland University Medical Centre, Kirrberger Str. Geb. 6.5, D-66421, Homburg/Saar, Germany; Fu Jen Catholic University, TAIWAN

## Abstract

**Background:**

The recently implemented mARC-rotation-technique is capable to deliver high dose rate bursts. For the case of hypopharynx cancer plans we evaluate whether the mARC can achieve an advantage in treatment time in comparison to IMRT. These plans consider two arcs with flat and flattening filter free (FFF) beam energies.

**Materials and Methods:**

For 8 hypopharynx-cancer patients step-and-shoot-IMRT and mARC plans were created retrospectively using flat and FFF beam energy. The comparison of the plan scenarios considered measures of quality for PTV coverage and sparing of organs at risk. All plans were irradiated on an anthromorphic phantom equipped with thermoluminescent dosimeters to measure scattered dose and treatment times.

**Results:**

A visual comparison of the dose distribution did not show a marked preference for either technique or energy. The statistical evaluation yielded significant differences in favor of the mARC technique and the FFF energy. Scattered dose could be decreased markedly by the use of the mARC technique. Treatment times could be reduced up to 3 minutes with the use of mARC in comparison to IMRT. The high dose rate energy results in another time advantage of about 1 minute.

**Conclusions:**

All four plan scenarios yielded equally good quality plans. A combination of the mARC technique with FFF 7 MV high dose rate resulted in a decrease of treatment times from about 9 minutes to 5–6 minutes in comparison to 6 MV IMRT.

## Introduction

Modern radiotherapy offers highly conformal treatment by using rotational techniques with the possibility to decrease treatment times due to continuous gantry rotation and MLC movement. There is a large body of literature evaluating VMAT (volumetric modulated arc therapy) and RapidArc for Elekta and Varian machines regarding plan quality and treatment times [1]. The analogue modulated arc (mARC) technique available for Siemens accelerators as a rotational hybrid IMRT (intensity modulated radiation therapy) technique is technically rather different from VMAT and has not been thoroughly evaluated so far.

Although first mARC applications have already been presented [2–4], only few studies have concentrated on the systematic evaluation of this technique. Previous planning studies [5,6] assessed the quality of mARC treatment as compared with IMRT for prostate cancer. In that study highly conformal treatment plans could be created both by the use of IMRT or mARC. It was possible to reduce treatment times from over 5 minutes to about half this value by using a combination of mARC with the flattening filter free (FFF) beam energy.

For comparatively simple target volumes, as it is in the case of the prostate without lymphatics, a single arc (one full gantry rotation) will yield good quality plans [7]. However, for more complicated target volumes (e.g. head and neck cancers), one arc may not be sufficient to create an optimal dose distribution. In those cases, a second arc can be included, with the potential consequence of losing the time advantage of mARC in comparison with IMRT [8].

In this planning study we therefore focus on a relatively complex target volume, hypopharyngeal carcinoma, to evaluate if the mARC technique can yield equally good quality plans. Moreover we analyse if there is an advantage in treatment time in comparison to IMRT although two full gantry rotations are needed. As the mARC can, but need not, be combined with a FFF beam energy, we include both a flat 6 MV and a FFF 7 MV photon beam in the analysis.

## Materials and Methods

### mARC technique

**Fig 1:**
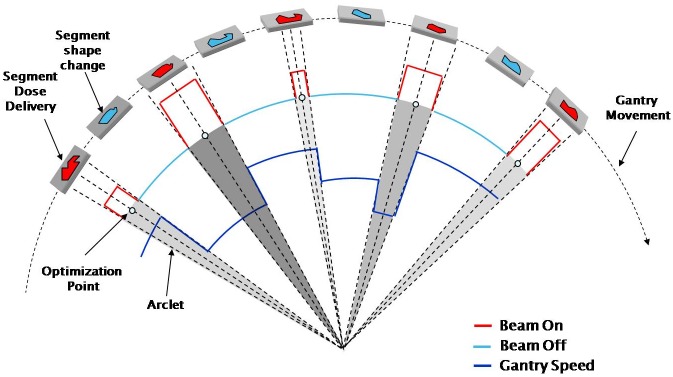
Schematic illustration of mARC delivery.

The mARC technique as a Siemens analogue to VMAT and RapidArc offers rotational hybrid IMRT irradiation in burst mode [2, 9]. Whereas RapidArc and VMAT are irradiated dynamically in a way such that the beam remains on (with or without varying the dose rate) while the gantry rotates around the patient and the MLC changes shape, the mARC technique pursues an intermediate approach: Here, phases of “beam on” are seperated from MLC movement while maintaining a continuous gantry rotation around the patient. Between two successive control points, as radiation is switched off, the MLC changes its configuration; after this (between the next two control points) the MLC configuration remains fixed and dose is delivered continuously over a small arclet (Fig 1). As a result, separate dose “bursts” are emitted over small arclets of generally 2–4° length during a continuous gantry rotation. This offers high dosimetric accuracy as the TPS (treatment planning system) usually calculates the dose based on static fields, which are solely “smeared” over a small angle, but no interpolation of MLC settings needs to be considered. In comparison to IMRT, a large number of beam angles and hence degrees of freedom are available for optimization while delivery times can be reduced due to continuous gantry rotation. In comparison to VMAT/RapidArc, the number of MLC segments appears slightly reduced in the final plan and irradiation phase, but it is acutally similar in the optimization—in fact, the SmartArc optimizer considers MLC segments every 8° and then interpolates in between, which implies the same degrees of freedom as for mARC optimization with 4° arclets spaced 4° apart. As a matter of fact, both kinds of plans can be converted into one another ([10]), but obviously no linac is capable of irradiating both. Due to the continuous irradiation, VMAT/RapidArc plans are theoretically slightly faster than mARC, where only half the rotation is used for irradiation, the other half to MLC movement—however, differences between the linacs equipped with VMAT/RapidArc vs. mARC preclude a direct comparison. On the other hand, mARC plans are not limited by the interaction of dose rate and MLC leaf speed, as the gantry rotation speed is automatically adjusted so that any desired MLC shape can be formed between successive arclets and any desired dose can be irradiated within each arclet (compare [11]).

By using a flattening filter free (FFF) mode, it is possible to irradiate with high dose-rate burst up to 2000 monitor units (MU) per minute. The beam profiles show the characteristic cone-shaped dose fall-off from the central axes in contrast to the uniform beam profiles of flat beams. To compensate for the spectral softening beam energy for the FFF mode is slightly decreased to 7 MV resulting in similar depth dose curves, energy spectrum and surface dose in comparison with flat 6 MV beams [12]. The mARC technique is hence particularly well adapted for high dose rate rotational treatment, as the dose rate does not need to be adapted to the available leaf speed as in volumetric modulated arc therapy (VMAT), which may be advantageous both from the point of view of plan quality and dosimetric accuracy.

### Patients´data collection and technical background

The present study is based on planning CT´s of 8 head and neck-cancer patients (four male, four female, 45–78 years of age) treated at our department. The patients, treated between October 2010 and September 2012, were selected from a previous planning study [13]. For the present study, the anonymized data from the previous study were used, with no further interaction with the patients. Therefore, an approval by the local ethics committee was not necessary due to the retrospective nature of this evaluation. The study was approved by our Institutional Review Board and formal written waiver for the need of ethics approval was issued by the departmental chair.

Planning target volume (PTV)-contours were drawn by a senior radiation oncologist for hypopharynx tumor sites. The PTV includes bilateral cervical and supraclavicular lymph nodes (level II-V). No boost-contours are included in this study because of greater individual variability; besides, the most extended and complex volume for planning is the PTV, which is therefore exclusively considered here. A total dose of 50 Gy (fraction dose 2 Gy) was prescribed so that 95% of the PTV received 95% of the prescribed dose.

At our department, one of the three available linear accelerators (linac), a Siemens Artiste machine, is equipped with the mARC technique and flat 6 MV as well as flattening filter free 7 MV beam energies. The MLC consists of 160 leafs with a width of 5 mm at isocenter distance.

Both energies show very similar depth dose characteristics, as the FFF 7 MV beam compensates for the spectral softening caused by the removal of the flattening filter [12]. Therefore a comparative planning study will reflect mainly the difference in beam profiles.

### Treatment planning

For IMRT plans 11 beams were used with about 5 segments per beam with the step-and-shoot-technique and 2 collimator orientations, based on the approach of a previous study [13] for hypopharynx cancer (gantry angles (collimator angle): 0° (90°), 30° (0°), 65° (0°), 100° (0°), 135° (90°), 170° (90°), 190° (90°), 225° (90°), 260° (0°), 295° (0°), 330° (0°)).

The same previous study showed that high quality IMRT plans could be achieved using 50–60 segments, which we consequently use as the “gold-standard” for head-and-neck IMRT planning at our institution. In individual cases, the segment number is reduced for the sake of decreasing treatment time if good plan quality can be maintained. Although many literature studies about IMRT planning rely on a smaller number of beams (and possibly also a smaller number of segments), the auto-sequencing option of the Artiste linac allows relatively fast irradiation because the gantry rotates automatically between the beams, so that employing 11 beams with a total of 55 segments is feasible in the clinical routine. Therefore, this plan scenario is taken as the baseline for comparison regarding plan quality of mARC plans.

The mARC plans involve two complete (360°) gantry rotations with optimization points every 8° and arclet length of 4° (88 control points). Hence, the mARC plans offer more degrees of freedom for the optimizer (45 optimization points per arc); however, we chose not to increase the number of segments for the IMRT plans, since plans with 90 segments are never used at our department due to the long treatment time. Moreover, hardly any improvement in plan quality is normally observed for plans exceeding 50–60 segments [13].

So for this study we evaluate four plan scenarios, two mARC plans with flat 6 MV and FFF 7 MV and the same for the IMRT technique.

Planning is performed in the Eclipse treatment planning system (TPS) V13.5 (Varian Medical Systems, Palo Alto, CA) on a 2.5 mm dose grid using an anisotropic-analytic-algorithm based on planning CT data from a Brilliance BigBore CT (Philips, Koninklijke, 3 mm spacing between slices).

Using a similar set of inversion objectives for both techniques, the optimization can be carried out interactively by adjusting the dose-volume-histogram (DVH) objectives (given in Table 1) and weights until the desired shape is reached. Plans were normalized so that 95% of the PTV volume was encompassed by 95% of the prescribed dose.

**Table 1:** DVH criteria for plan acceptability.

PTV	V(95%) > 95%
	V(105%) < 5%
Spinal cord	D_max_ < 27 Gy
Extended spinal cord (+ 5 mm)	D_max_ < 33 Gy
Parotid glands	V(14Gy) < 30%

Maximum objectives for the organs at risk were scaled down since this planning study disregarded subsequent boost irradiation up to a total of 76 Gy. Although the maximum dose to the spinal cord and extended spinal cord are given as objectives, the final assessment of the plans relies on the dose to a small volume (ca. 1%) rather than the point maximum.

### Statistical analysis and evaluation

All plans (DVHs and dose distributions) were reviewed by a senior radiation oncologist and a visual comparison of the plan qualities for the four scenarios (IMRT vs. mARC, 6 MV vs. 7 MV) was conducted.

For the statistical evaluation, we consider the quality indices for PTV conformity (CI) [14] and homogeneity (HI):

$$CI=\frac{{\left(TV_{PIV}\right)}^{2}}{TV\cdot PIV}$$

where *TV* denotes the volume of the PTV, *PIV* is the volume of the prescribed isodose (95%) and *TV*_*PIV*_ is the volume of the PTV covered by the prescribed isodose (95%);

$$HI=\frac{D_{PTV}(2\%)-D_{PTV}(98\%)}{D_{PTV}(50\%)}$$

where D_PTV_(X%) is the dose received by X% of the PTV volume.

Sparing of organs at risk is assessed by DVH-objectives for the parotid glands (V(20%) and mean) and the spinal cord (D(1%) and D(2%)).

All plans were exported to the machine for treatment and irradiated on an anthromorphic Alderson phantom to record treatment times. The phantom was equipped with thermoluminescent dosimeters (TLD, Harshaw TLD 100H, Thermo Scientific, United States, Massachusettes) at the left breast (about 25 cm distance to the isocenter) to measure scattered out-of-field dose. Three TLDs were placed in close proximity and the readings were averaged. Readout was performed using a Harshaw TLD 5500 Automatic Reader.

The statistical analysis was performed using a two-way ANOVA with repeated measures and interactions. Plans were compared pairwise with the Holm-Bonferroni test for mARC vs. IMRT and for 6 MV vs. FFF 7 MV energies, considering the measures of quality defined above, monitor units (MU), treatment times and scattered dose.

## Results

**Fig 2:**
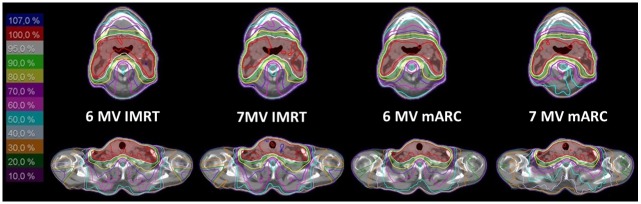
Example dose distributions for the four plan scenarios (transverse slice).

**Fig 3:**
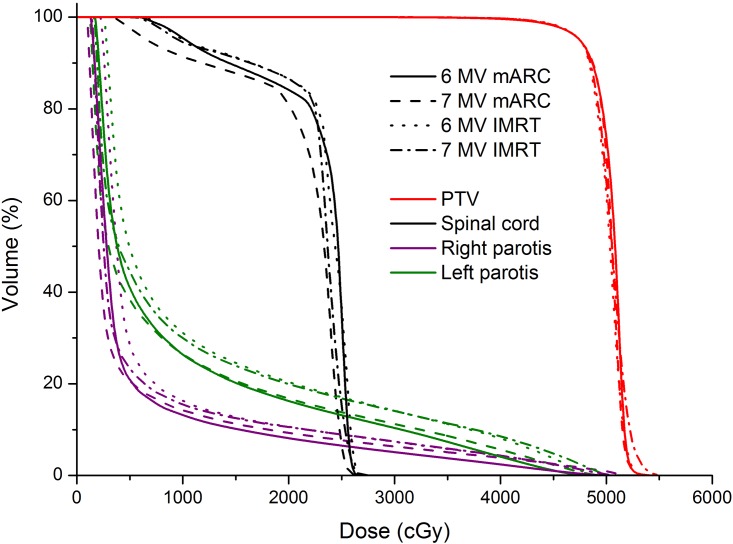
Example DVH (same patient as in Fig 2).

Based on an inspection of the dose distributions and DVHs, the radiation oncologist approved all plans acceptable for treatment (examples shown in Figs 2 and 3).

All plans satisfy the criteria for PTV coverage and sparing of OAR (Table 1). A visual comparison of the four plan scenarios for each patient generally did not show a marked preference for either technique or energy (Figs 2 and 3, which are representative for the whole patient collective). In some cases the IMRT plans reaches a better sparing of the spinal cord, whereas the dose distribution of mARC plans shows a higher conformity in PTV coverage, especially in the area of the oral cavity. This result also becomes apparent in the statistical evaluation, mARC plans reaching significantly higher conformity than the corresponding IMRT plans (6MV: mARC 0.85 vs IMRT 0.78, p = 4.9e-4, 7 MV: mARC 0.86 vs IMRT 0.78, p = 4.5e-4) while the comparison of the two energies showed no differences (Tables 2 and 3). The homogeneity index is suggestive of yielding better results for mARC FFF 7 MV in comparison to mARC 6 MV and for IMRT 6 MV in comparison to mARC 6 MV; however, looking at the absolute values, it can be seen that the differences are very small (average 0.16 vs. 0.17) and probably lack clinical relevance.

**Table 2:** Measures of quality for the four plan scenarios (mean values ± standard deviation and range).

Index	6 MV IMRT	7 MV FFF IMRT	6 MV mARC	7 MV FFF mARC
PTV D50% (Gy)	50.44±0.25 (49.9–50.7)	50.65±0.2 (50.3–50.9)	50.83±0.51 (50.0–51.5)	50.49±0.46 (49.8–51.1)
CI	0.78±0.04 (0.69–0.81)	0.78±0.02 (0.75–0.8)	0.85±0.02 (0.83–0.88)	0.86±0.02 (0.82–0.88)
HI	0.16±0.019 (0.14–0.18)	0.17±0.013 (0.14–0.18)	0.17±0.02 (0.14–0.2)	0.16±0.03 (0.11–0.19)
Spinal cord D1% (Gy)	24.99±1.84 (22.2–26.7)	24.88±1.75 (22.1–26.3)	26.25±0.53 (25.4–27.0)	25.66±0.46 (24.8–26.1)
Spinal cord D2% (Gy)	24.67±1.91 (21.84–26.5)	24.6±1.84 (21.7–26.1)	26.04±0.44 (25.3–26.6)	25.47±0.49 (24.6–26)
Parotid mean (Gy)	12.96±1.98 (7.85–15.25)	12.36±2.12 (6.95–14.8)	12.23±2.52 (6.1–14.45)	11.36±2.01 (6.15–13.1)
Parotid V20% (Gy)	18.60±3.92 (10.6–24.4)	18.07±3.33 (10.5–23.54)	17.80±3.57 (8.12–21.24)	16.65±2.88 (9.3–20.03)

CI: Conformity Index, HI: Homogeneity Index.

**Table 3:** Results of the two-way anova with repeated measures and interactions (p-values of pairwise tests for the measures of quality).

Index	Energy	Technique	Interaction	p(6 MV IMRT—mARC)	p(7 MV FFF IMRT—mARC)	p(IMRT 6 MV– 7 MV FFF)	p(mARC 6 MV– 7 MV FFF)
PTV D50% (Gy)	n.s.	n.s.	0.0074	0.019	n.s.	n.s.	0.034
CI	n.s.	1.6e-4	n.s.	4.9e-4	4.5e-4	n.s.	n.s.
HI	n.s.	n.s.	0.026	0.046	n.s.	n.s.	0.032
Spinal cord D1% (Gy)	n.s.	n.s.	n.s.	n.s.	n.s.	n.s.	n.s.
Spinal cord D2% (Gy)	n.s.	n.s.	n.s.	n.s.	n.s.	n.s.	n.s.
Parotid mean (Gy)	4.8e-5	0.0062	n.s.	0.015	0.0025	0.037	0.0058
Parotid V20% (Gy)	0.00973	n.s.	n.s.	n.s.	0.027	n.s.	n.s.

n.s.: not significant, CI: Conformity Index, HI: Homogeneity Index.

Since the plan normalization is performed so that 95% of the prescribed dose (50 Gy) covers 95% of the PTV, the coverage is obviously identical in all plans. In addition, Table 2 shows that for all scenarios the PTV D50% objective results in values near to the prescribed dose (50 Gy) and the four scenarios differ by less than 0.5 Gray (6 MV: IMRT 50.44 Gy vs. mARC 50.65 Gy, 7 MV: IMRT 50.83 Gy vs. mARC 50.49 Gy).

The statistical evaluation of OAR sparing mostly shows no significant differences (e.g. spinal cord D1%: 6 MV: IMRT 24.99 Gy vs mARC 26.25 Gy). In those cases where a statistically significance is found, it is always in favor of the mARC technique and the FFF 7 MV energy (e.g. parotid mean: IMRT: 6 MV 12.96 Gy vs 7 MV 12.36 Gy, p = 0.037, mARC: 6MV 12.23 Gy vs 7 MV 11.36 Gy, p = 0.0058).

The use of the FFF 7 MV beam energy results in considerably more monitor units than the flat 6 MV. For 7 MV, IMRT and mARC require almost the same amount of MUs, the IMRT FFF 7 MV plans need about 779 MU in comparison to 758 MU for the corresponding 7 MV mARC plans. 6 MV IMRT require 598 MUs on average, whereas mARC 6 MV required the fewest MUs, with an average of about 406.

Treatment times could be markedly decreased by the use of the high dose rate and the mARC technique (Tables 4 and 5). IMRT-plans needed about 9 minutes with the flat energy and 7–8 minutes using FFF 7 MV. The times for the mARC plans, each with two full gantry rotations, range between 6 and 7 minutes for the flat energy and 5–6 minutes for the FFF energy.

**Table 4:** Monitor units, treatment times and scattered dose for the four plan scenarios (mean values ± standard deviation and range).

Index	6 MV IMRT	7 MV FFF IMRT	6 MV mARC	7 MV FFF mARC
Monitor units	598±89 (485–729)	779±95 (628–918)	406±32 (363–459)	758±92 (644–897)
Treatment times (min:sec)	9:09±0:32 (8:33–10:09)	7:46±0:14 (7:22–8:08)	6:19±0:06 (6:09–6:29)	5:30±0:04 (5:25–5:37)
Dose at breast (mGy)	24.1±2.63 (20.4–27.7)	20.5±3.12 (16.8–25.7)	17.9±4.13 (13.4–22.6)	15.4±2.87 (12.3–18.4)

**Table 5:** Results of the two-way anova with repeated measures and interactions (p-values of pairwise tests for monitor units, treatment times and scattered dose).

Index	Energy	Technique	Interaction	p(6 MV IMRT—mARC)	p(7 MV FFF IMRT—mARC)	p(IMRT 6 MV– 7 MV FFF)	p(mARC 6 MV– 7 MV FFF)
Monitor units	1.79e-6	0.00223	0.0124	5.1e-4	n.s.	7.6e-4	1.09e-5
Treatment times (min:sec)	1.36e-5	5.75e-8	0.0447	3.2e-7	1.4e-6	4.3e-5	0.00108
Dose at breast (mGy)	1.73e-5	7.78e-6	n.s.	1.2e-5	4.3e-5	3.98e-4	0.00303

n.s.: not significant

Scattered dose was considerably lower (25% - 26%) for the mARC as compared to IMRT (Tables 4 and 5). The comparison of 6 MV vs. FFF 7 MV shows a reduction of about 15% with the use of the high dose rate (6 MV: IMRT 24.1mGy vs mARC 17.9 mGy, 7 MV: IMRT 20.5 mGy vs mARC 15.4 mGy).

## Discussion

The statistical evaluation confirms the results of the visual inspection of DVH and dose distributions. All four scenarios (IMRT 6 MV and 7MV, mARC 6 MV and 7 MV) lead to comparatively good quality plans. If there are significant differences related to homogeneity or conformity, the absolute values of CI and HI show little differences, resulting in reduced clinical relevance. These findings are similar to the results obtained in Mynampati DK et al. [15] comparing IMRT plans with VMAT plans in Eclipse.

The sparing of organs at risk also shows no significant differences in most cases. Looking at the few cases where significant differences exist, they are always in favor of the mARC-technique and the FFF 7 MV energy. However, in these cases as well, absolute differences are so small to lack clinical relevance.

Monitor units differ significantly between 6 MV and FFF 7 MV. This is plausible because of the large PTV in head and neck cases. The dose intensity of FFF beams decreases with distance from the central axis, so with a large PTV more MUs are needed to add dose far from the central axis. This does not occur for the flat energy, because the dose profile does not peak and can cover a larger part of the PTV uniformly with less MUs. These findings were also observed in the study by Dzierma et al. [13].

In spite of this, flattening-filter-free beam energies result in a reduction of treatment time. Therefore, the most efficient way to reduce treatment times is to combine the mARC technique with the high dose-rate FFF 7 MV energy, with a time advantage up to factor 2 in comparison to IMRT 6 MV.

The absence of the flattening filter physically leads to a reduction in scattered dose per MU. This has also been observed in other studies [5, 16–20]. However, for complex target volumes this effect competes with the higher amount of monitor units that are needed for the flattening filter free treatment plans, so that it is not *a priori* clear which effect will be dominant. Although the number of monitor units is markedly elevated, our study shows that scattered dose at the breast is still decreased with the use of FFF 7 MV.

The reduction in scattered dose for mARC in comparison to IMRT is supposed to be caused by the fact that Y jaws were completely opened for IMRT within the Eclipse TPS. In contrast to that the TPS adapts the Y jaws to the maximum opening of the treatment field for mARC, leading to less leakage radiation. Similar findings occurred for the previous study regarding prostate planning [6].

The biological aspects of the high dose rate are discussed controversially. While in most studies no effects of the instantaneous dose rate on cell survival is observed *in vitro* [21–23], one study reports a statistically significant reduction of clonogenic survival for higher dose rate [24]. However, investigations on this topic are limited and there is a need of more data, especially *in vivo*. As mARC and IMRT are modulated techniques, treatment times remain of the order of a few minutes and the delivered dose per fraction is much smaller in comparison to stereotactic treatment, so the relevance of potential biological effects of the high dose rate may be questionable.

### Comparison with previous studies

Kainz et al. [2] have been one of the first to examine mARC-treatment, although they include just one example for a head-and-neck indication. For the right piriform sinus plus cervical lymph nodes, they present a plan consisting of a single arc with 2 Gy for the primary PTV and 1.54 Gy for the lymph nodes, respectively, in 35 fractions. Using 36 optimization points they observed a delivery time for this plan of 4 minutes. As only one arc was used, this is not quite comparable to this study. Another mARC-planning study for Eclipse TPS [25] included two head and neck cases with two arcs, respectively. With a prescription of 2.25 Gy per fraction (6 MV) the mARC-plans required about 10 minutes and 699 and 518 MUs respectively. In comparison to our study, treatment times were considerably longer and more monitor units were required. As it is not exactly described which kind of head and neck cancer was considered in their study, the differences may result from different target volumes. Moreover the choice of the arclet spacing might be different from ours.

In previous studies [5,6], mARC vs IMRT with flat and flattening-filter-free beam energies for prostate cancer patients were compared. In these planning studies highly conformal treatment plans could be created for all modalities using one full gantry rotation. For the Eclipse TPS treatment times could be decreased by a factor of three for the combination of the mARC-technique with the FFF 7 MV beams in comparison to IMRT 6 MV (2:30 for mARC 7 MV, 7:16 for IMRT 6 MV) [6]. For a transition to more complex target volumes like head and neck cases, two full gantry rotations may be needed to achieve sufficient dose distributions. The additional rotation causes an increase in treatment times. However, as this study shows, even though two rotations are needed, the mARC-technique still results in shorter treatment times in comparison to a corresponding IMRT, without detriment of plan quality. In our present study, the time required to irradiate two arcs in FFF mode is 5:30 min on average, which agrees well with the previous study showing irradiation time of carcinoma prostate of ca. 2:30 min for a single arc (FFF energy). Given the fact that about 20 seconds elapse between the end of the first rotation and the beginning of the second, it is evident that the gantry rotation speed is closely similar in both scenarios.

Regarding VMAT, treatment times differ between different linacs and planning approaches. Several planning studies for head and neck cancer result in about 3–4 min for two complete gantry rotations using a flattening filter [7, 8, 26]. Other studies [27] report 5:30 min in the absence of a flattening filter, which is similar to the delivery times of the FFF plans of our present study.

### Limitations

In the context of a scientific cooperation, we were able to use the Eclipse TPS only for a limited period of time for the evaluation of the mARC planning option. Consequently the number of cases presented here is rather small. Moreover, the use of a simultaneous integrated boost (SIB) concept for head and neck treatment is presently considered [28, 29], which could bring up new aspects and challenges for this topic. A final point is the dependence of the planning workflow and results on the experience of the physicist or dosimetrist. We tried to avoid this issue by using the same inversion objectives for all plans to offer equal conditions for comparison, but clinical reality is that every plan is individually planned and adjusted to the single patients.

## Conclusion

For all modalities (IMRT vs mARC, flat 6 MV vs FFF 7 MV) it was possible to create highly conformal treatment plans. In those cases where significant differences exist, they are mostly in favor of the mARC technique and the FFF 7 MV energy. Monitor units and hence integral dose are considerably higher for the FFF 7 MV than the flat 6 MV energy; they are reduced by the use of mARC.

Although two full gantry rotations are needed for hypopharynx treatment, it is possible to reduce treatment times by up to three minutes by using the mARC technique instead of IMRT. The use of the high dose rate energy results in another time advantage of about one minute.

Consequently, with a combination of the mARC technique with FFF 7 MV high dose rate, treatment times could be reduced from about 9 minutes to 5–6 minutes in comparison to 6 MV IMRT for hypopharynx treatment, while scattered dose was reduced and plan quality remained comparable, or even better.

## Supporting Information

S1 Fig DVH of Patient 1. (TIF)

S2 Fig DVH of Patient 2. (TIF)

S3 Fig DVH of Patient 3. (TIF)

S4 Fig DVH of Patient 4. (TIF)

S5 Fig DVH of Patient 5. (TIF)

S6 Fig DVH of Patient 6. (TIF)

S7 Fig DVH of Patient 7. (TIF)

S8 Fig DVH of Patient 8. (TIF)

S1 Table Individual data points of the measurements for all patients. (DOCX)
