# Supplementary figures and images for: mARC Treatment of Hypopharynx Carcinoma with Flat and Flattening-Filter-Free Beam Energies – A Planning Study
Source: PLoS One. 2016 Oct 14;11(10):e0164616. doi: 10.1371/journal.pone.0164616 (PMC5065169; doi:10.1371/journal.pone.0164616)

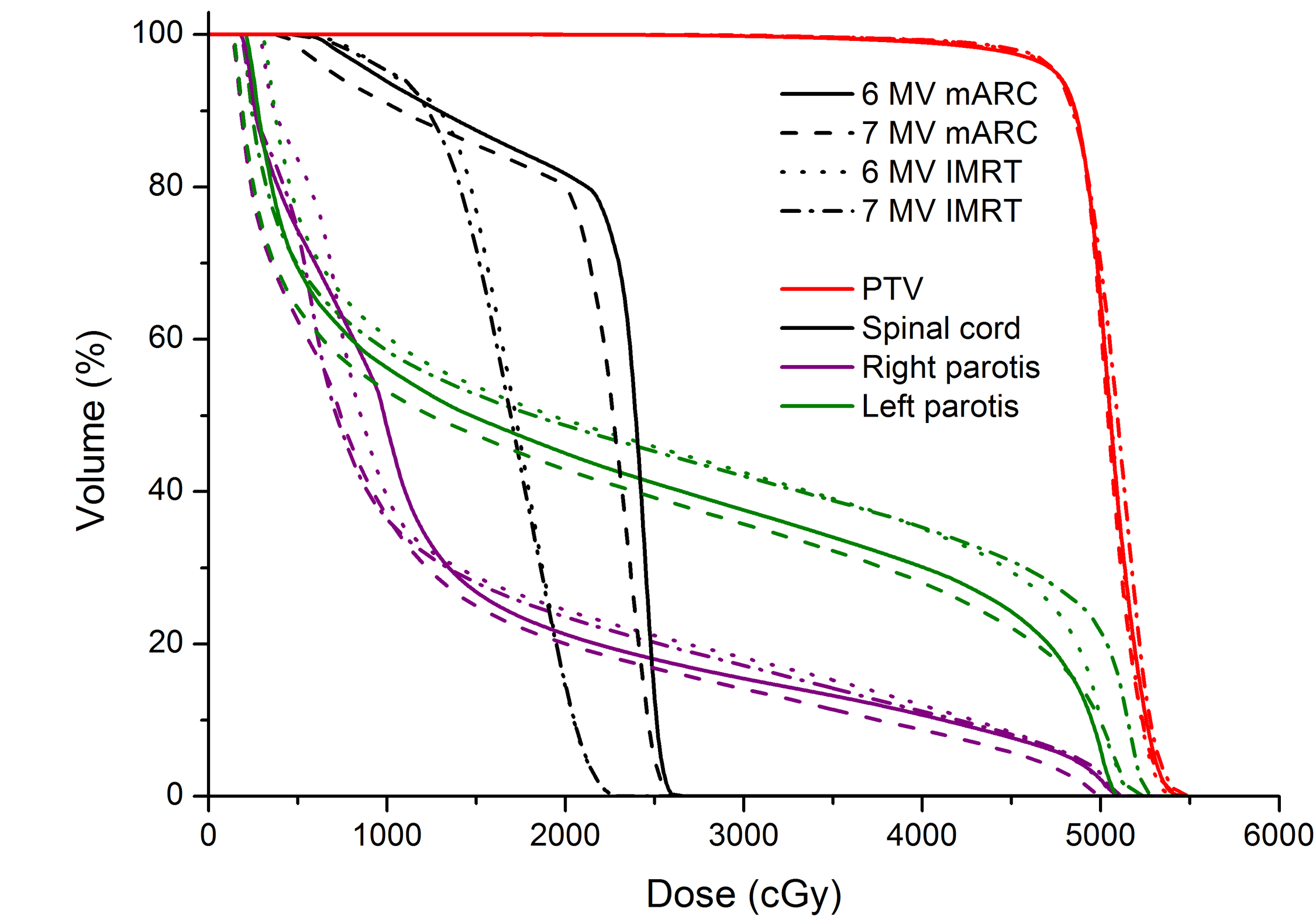

Supplement: S1 Fig — (TIF) [file pone.0164616.s001.tif]

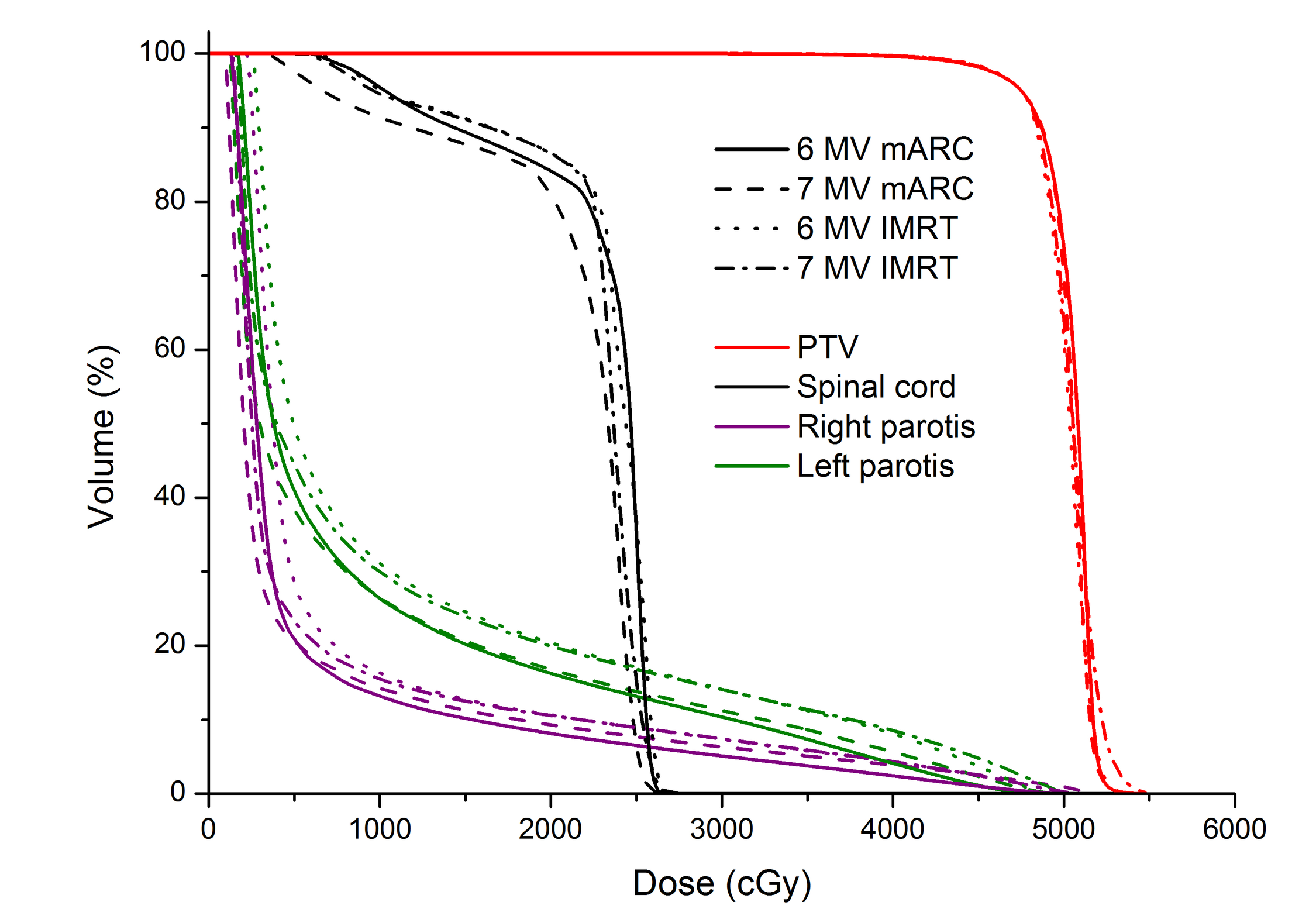

Supplement: S2 Fig — (TIF) [file pone.0164616.s002.tif]

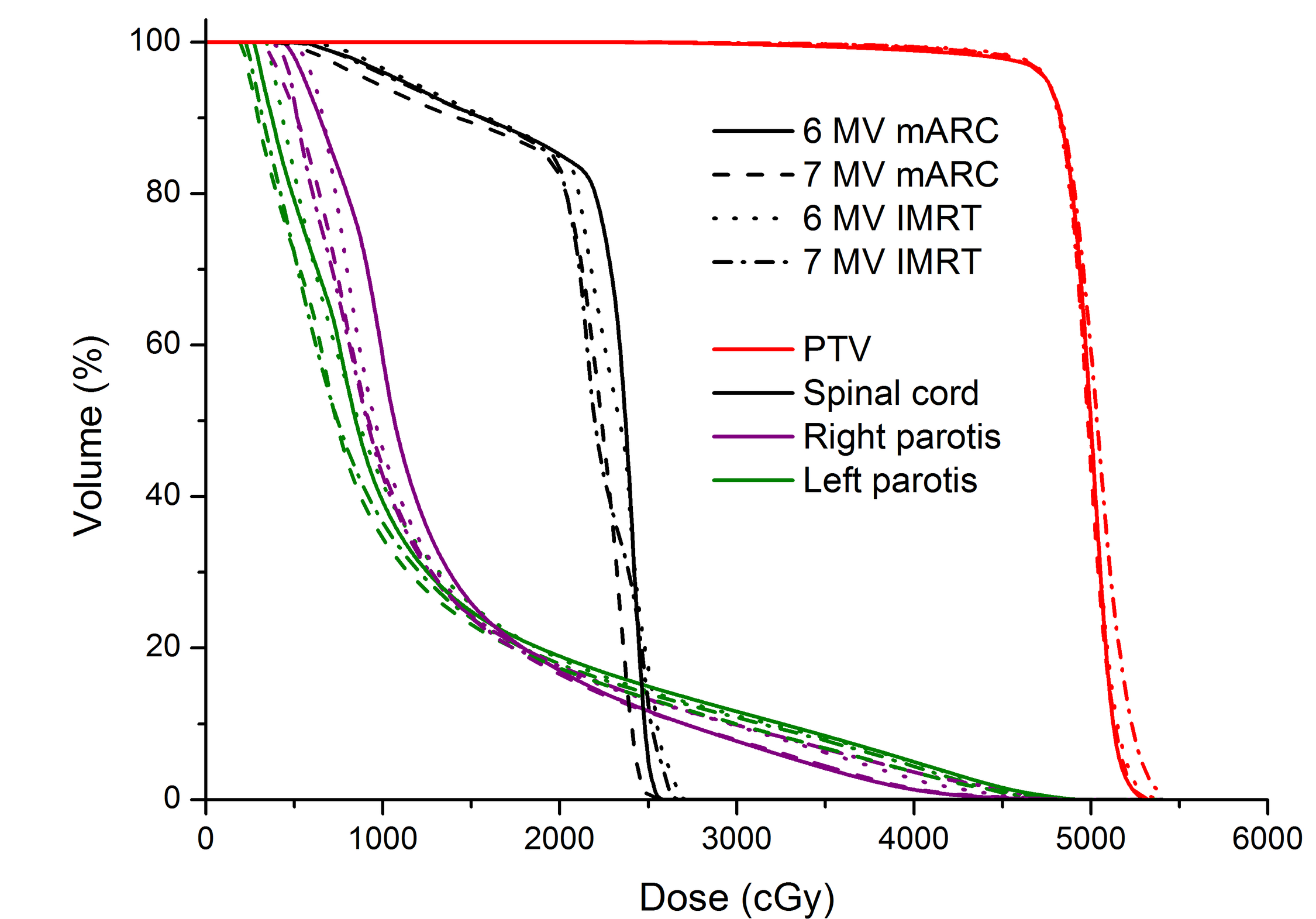

Supplement: S3 Fig — (TIF) [file pone.0164616.s003.tif]

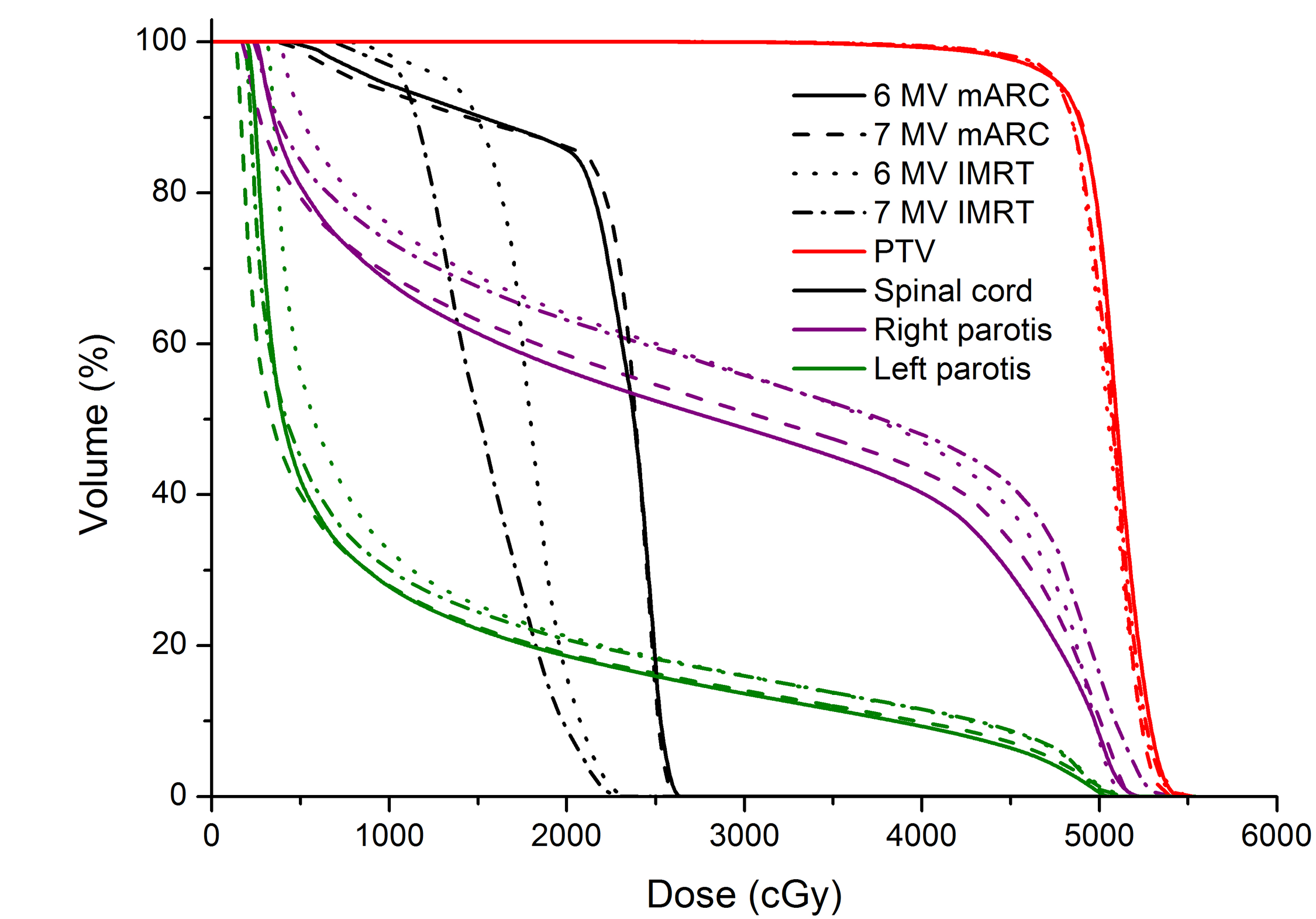

Supplement: S4 Fig — (TIF) [file pone.0164616.s004.tif]

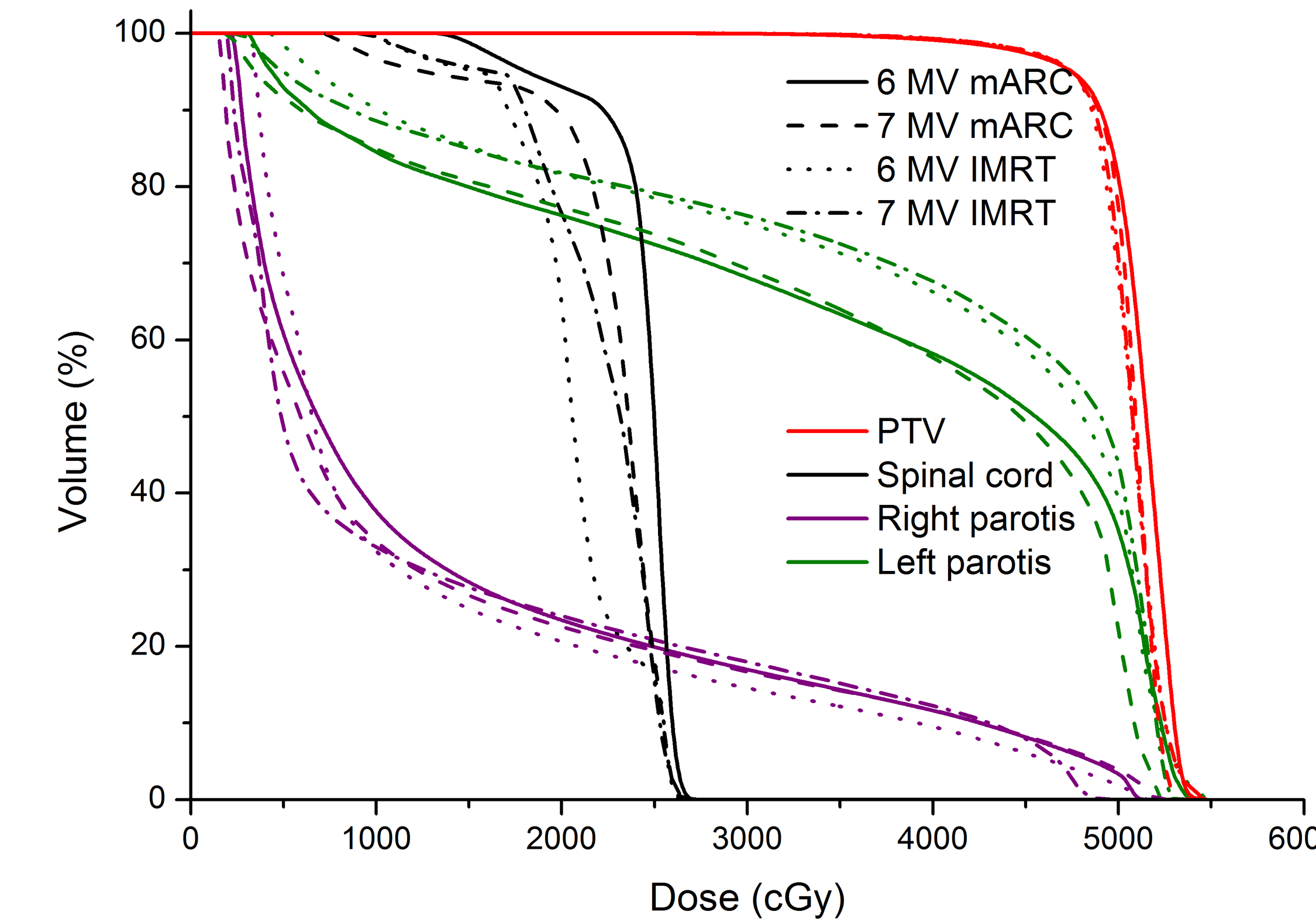

Supplement: S5 Fig — (TIF) [file pone.0164616.s005.tif]

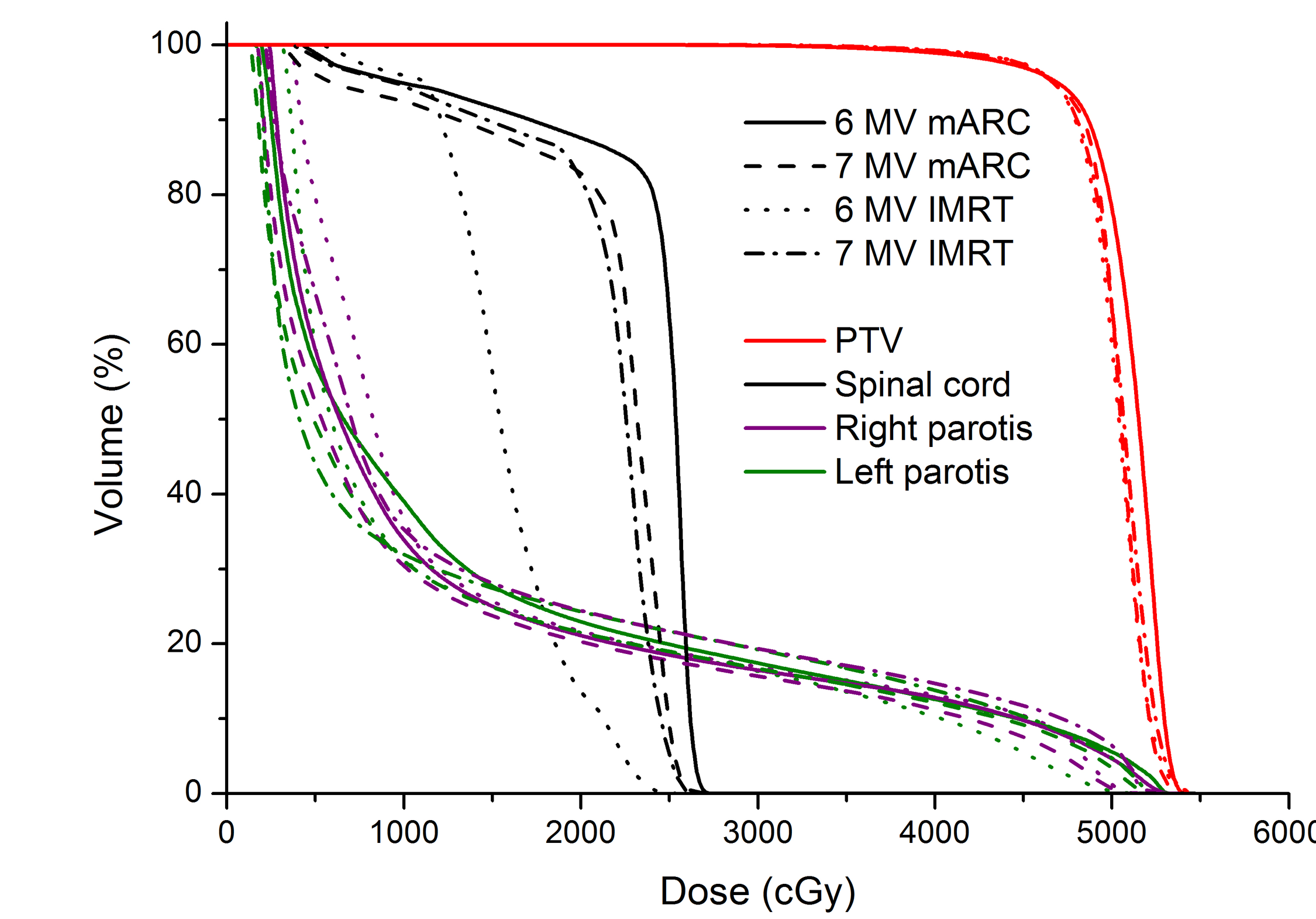

Supplement: S6 Fig — (TIF) [file pone.0164616.s006.tif]

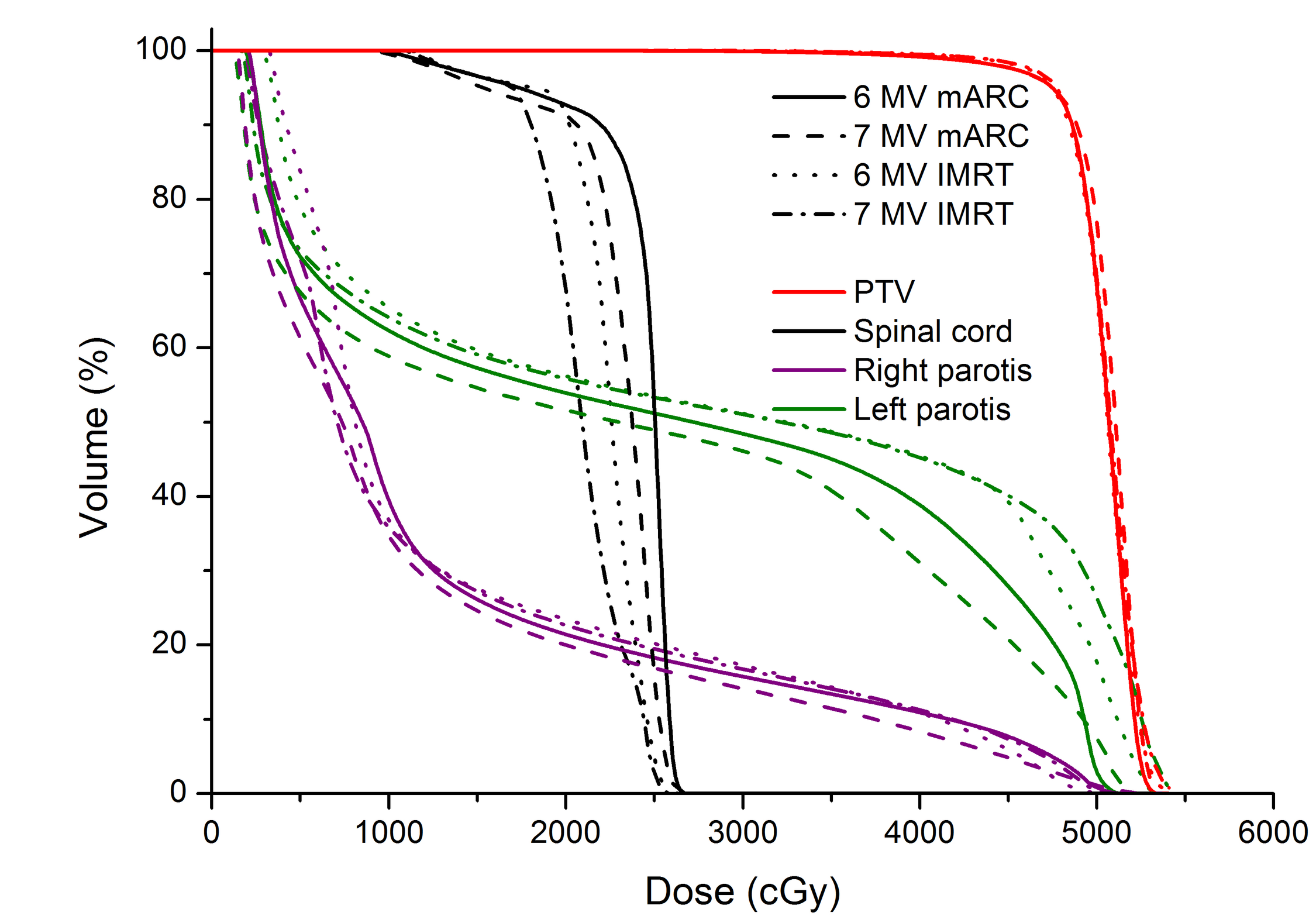

Supplement: S7 Fig — (TIF) [file pone.0164616.s007.tif]

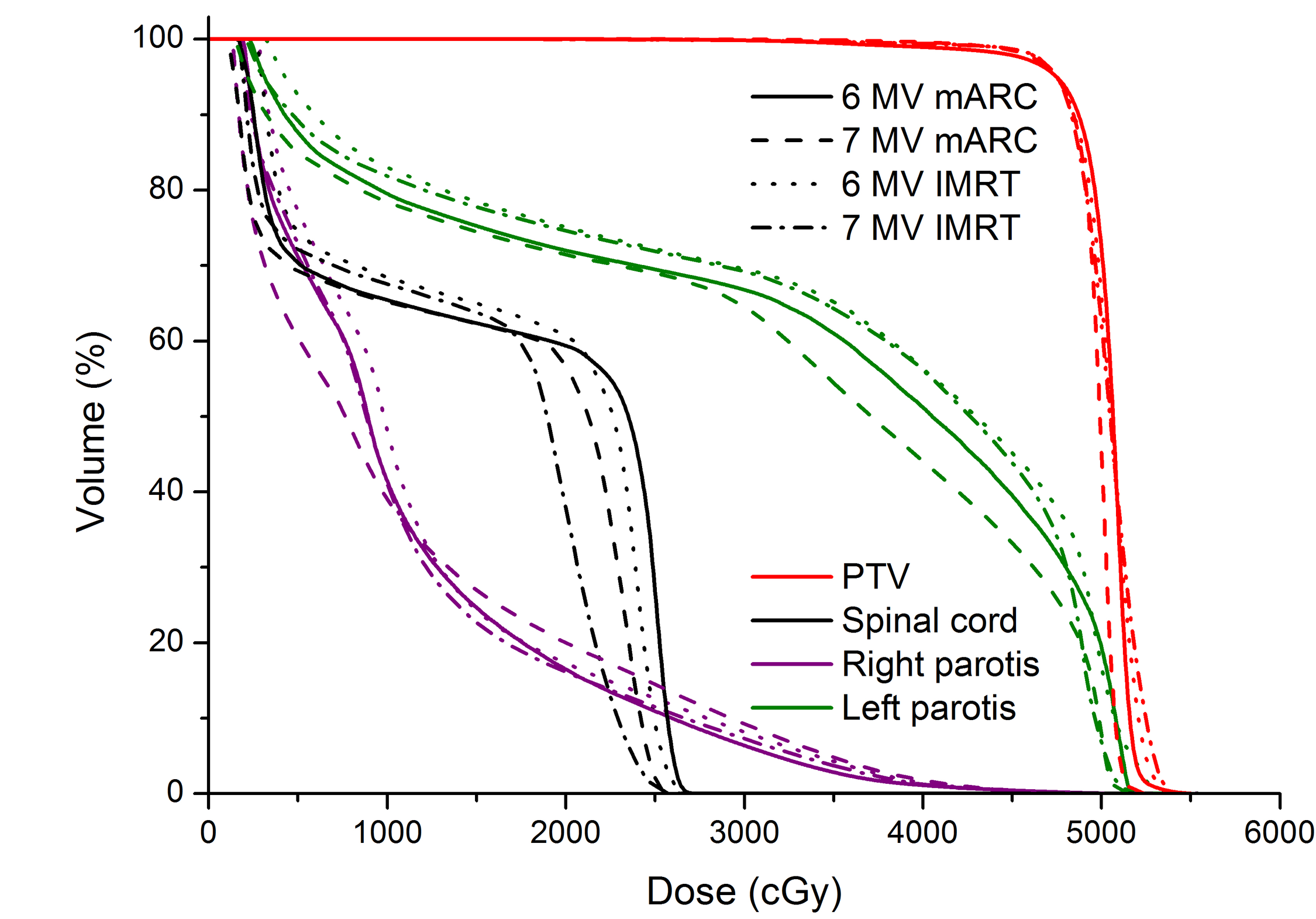

Supplement: S8 Fig — (TIF) [file pone.0164616.s008.tif]
