# Supplementary material for: mARC Treatment of Hypopharynx Carcinoma with Flat and Flattening-Filter-Free Beam Energies – A Planning Study
Source: PLoS One. 2016 Oct 14;11(10):e0164616. doi: 10.1371/journal.pone.0164616 (PMC5065169; doi:10.1371/journal.pone.0164616)
Supplement: S1 Table — (DOCX) [file pone.0164616.s009.docx]

**S1 Table. Individual data points of the measurements for all patients**

| PTV D50% [Gy] | IMRT-6MV | IMRT-7MV | mARC-6MV | mARC-7MV |
| --- | --- | --- | --- | --- |
| Patient 1 | 50.6 | 50.9 | 50.5 | 50.4 |
| Patient 2 | 50.3 | 50.5 | 50.8 | 50.5 |
| Patient 3 | 49.9 | 50.3 | 50.0 | 49.8 |
| Patient 4 | 50.5 | 50.7 | 51.0 | 50.8 |
| Patient 5 | 50.7 | 50.8 | 51.5 | 50.9 |
| Patient 6 | 50.4 | 50.7 | 51.5 | 50.5 |
| Patient 7 | 50.6 | 50.8 | 50.7 | 51.1 |
| Patient 8 | 50.5 | 50.5 | 50.6 | 49.9 |

| Conformity Index | IMRT-6MV | IMRT-7MV | mARC-6MV | mARC-7MV |
| --- | --- | --- | --- | --- |
| Patient 1 | 0.69 | 0.78 | 0.86 | 0.86 |
| Patient 2 | 0.8 | 0.79 | 0.87 | 0.87 |
| Patient 3 | 0.77 | 0.76 | 0.88 | 0.88 |
| Patient 4 | 0.81 | 0.79 | 0.86 | 0.86 |
| Patient 5 | 0.8 | 0.75 | 0.84 | 0.83 |
| Patient 6 | 0.78 | 0.8 | 0.84 | 0.82 |
| Patient 7 | 0.8 | 0.78 | 0.85 | 0.86 |
| Patient 8 | 0.78 | 0.78 | 0.83 | 0.86 |

| Homogeneity Index | IMRT-6MV | IMRT-7MV | mARC-6MV | mARC-7MV |
| --- | --- | --- | --- | --- |
| Patient 1 | 0.17 | 0.17 | 0.19 | 0.18 |
| Patient 2 | 0.14 | 0.16 | 0.14 | 0.14 |
| Patient 3 | 0.14 | 0.17 | 0.16 | 0.14 |
| Patient 4 | 0.18 | 0.16 | 0.18 | 0.17 |
| Patient 5 | 0.18 | 0.18 | 0.19 | 0.17 |
| Patient 6 | 0.18 | 0.18 | 0.2 | 0.19 |
| Patient 7 | 0.15 | 0.16 | 0.17 | 0.17 |
| Patient 8 | 0.14 | 0.14 | 0.15 | 0.11 |

| Spinal cord D1% [Gy] | IMRT-6MV | IMRT-7MV | mARC-6MV | mARC-7MV |
| --- | --- | --- | --- | --- |
| Patient 1 | 22.2 | 22.2 | 25.8 | 25.6 |
| Patient 2 | 26.4 | 26.3 | 26.1 | 25.7 |
| Patient 3 | 26.7 | 26.2 | 25.4 | 24.8 |
| Patient 4 | 22.6 | 22.1 | 26.0 | 25.9 |
| Patient 5 | 26.4 | 26.3 | 27.0 | 26.1 |
| Patient 6 | 23.8 | 25.7 | 26.8 | 25.9 |
| Patient 7 | 25.6 | 25.3 | 26.4 | 26.1 |
| Patient 8 | 26.2 | 24.9 | 26.5 | 25.2 |

| Spinal cord D2% [Gy] | IMRT-6MV | IMRT-7MV | mARC-6MV | mARC-7MV |
| --- | --- | --- | --- | --- |
| Patient 1 | 21.84 | 21.8 | 25.62 | 25.34 |
| Patient 2 | 26.3 | 26.1 | 25.9 | 25.4 |
| Patient 3 | 26.5 | 26.1 | 25.3 | 24.6 |
| Patient 4 | 22.2 | 21.7 | 25.9 | 25.7 |
| Patient 5 | 25.9 | 26.0 | 26.5 | 26.0 |
| Patient 6 | 23.3 | 25.5 | 26.6 | 25.7 |
| Patient 7 | 25.4 | 25.1 | 26.2 | 26.0 |
| Patient 8 | 25.9 | 24.5 | 26.3 | 25.0 |

| Parotid mean [Gy] | IMRT-6MV | IMRT-7MV | mARC-6MV | mARC-7MV |
| --- | --- | --- | --- | --- |
| Patient 1 left |  |  |  |  |
| right | 15.3 | 14.2 | 14.5 | 12.6 |
| Patient 2 left | 11.9 | 11.2 | 9.6 | 9.5 |
| right | 7.9 | 7.0 | 6.1 | 6.2 |
| Patient 3 left | 13.0 | 12.0 | 12.9 | 11.7 |
| right | 13.5 | 13.1 | 13.7 | 12.6 |
| Patient 4 left | 13.2 | 12.1 | 11.2 | 10.9 |
| right |  |  |  |  |
| Patient 5 left |  |  |  |  |
| right | 13.1 | 13.2 | 13.9 | 13.1 |
| Patient 6 left | 13.0 | 13.3 | 14.0 | 12.6 |
| right | 14.8 | 14.8 | 13.5 | 12.3 |
| Patient 7 left |  |  |  |  |
| right | 14.6 | 13.8 | 13.8 | 12.4 |
| Patient 8 left |  |  |  |  |
| right | 12.4 | 11.4 | 11.5 | 11.3 |

(Due to inappropriate anatomical positions it was not always possible to spare both parotids, so for some patients just one parotid was assessed)

| Parotid V20% [Gy] | IMRT-6MV | IMRT-7MV | mARC-6MV | mARC-7MV |
| --- | --- | --- | --- | --- |
| Patient 1 left |  |  |  |  |
| right | 24.4 | 23.5 | 21.2 | 20.0 |
| Patient 2 left | 20.4 | 19.9 | 16.3 | 16.8 |
| right | 10.6 | 10.5 | 8.1 | 9.3 |
| Patient 3 left | 18.5 | 17.9 | 18.9 | 17.3 |
| right | 17.1 | 17.6 | 17.0 | 16.4 |
| Patient 4 left | 21.2 | 20.8 | 18.6 | 18.9 |
| right |  |  |  |  |
| Patient 5 left |  |  |  |  |
| right | 16.2 | 16.5 | 18.8 | 16.8 |
| Patient 6 left | 15.6 | 16.0 | 19.6 | 15.6 |
| right | 18.2 | 17.6 | 16.9 | 15.6 |
| Patient 7 left |  |  |  |  |
| right | 18.4 | 17.9 | 19.7 | 17.3 |
| Patient 8 left |  |  |  |  |
| right | 24.0 | 20.5 | 20.7 | 19.5 |

(Due to inappropriate anatomical positions it was not always possible to spare both parotids, so for some patients just one parotid was assessed)

| Monitor Units | IMRT-6MV | IMRT-7MV | mARC-6MV | mARC-7MV |
| --- | --- | --- | --- | --- |
| Patient 1 | 546 | 918 | 423 | 795 |
| Patient 2 | 485 | 628 | 363 | 644 |
| Patient 3 | 524 | 688 | 419 | 792 |
| Patient 4 | 684 | 877 | 459 | 738 |
| Patient 5 | 661 | 816 | 400 | 694 |
| Patient 6 | 729 | 748 | 416 | 897 |
| Patient 7 | 633 | 801 | 405 | 851 |
| Patient 8 | 525 | 753 | 364 | 654 |

| Treatment times [min:sec] | IMRT-6MV | IMRT-7MV | mARC-6MV | mARC-7MV |
| --- | --- | --- | --- | --- |
| Patient 1 | 8:47 | 8:08 | 6:10 | 5:25 |
| Patient 2 | 8:41 | 7:56 | 6:22 | 5:26 |
| Patient 3 | 8:33 | 7:22 | 6:21 | 5:28 |
| Patient 4 | 9:19 | 7:44 | 6:29 | 5:28 |
| Patient 5 | 8:53 | 7:54 | 6:18 | 5:32 |
| Patient 6 | 9:29 | 7:33 | 6:20 | 5:33 |
| Patient 7 | 9:20 | 7:51 | 6:19 | 5:37 |
| Patient 8 | 10:09 | 7:45 | 6:09 | 5:28 |

| Dose at breast [mGy] | IMRT-6MV | IMRT-7MV | mARC-6MV | mARC-7MV |
| --- | --- | --- | --- | --- |
| Patient 1 | 25.3 | 22.6 | 21.8 | 18.4 |
| Patient 2 | 27.7 | 22.4 | 22.6 | 18.0 |
| Patient 3 | 20.4 | 16.8 | 13.4 | 12.3 |
| Patient 4 | 22.8 | 20.0 | 14.1 | 12.3 |
| Patient 5 | 27.1 | 25.7 | 22.3 | 18.0 |
| Patient 6 | 23.5 | 17.8 | 14.6 | 13.8 |
| Patient 7 | 24.9 | 21.6 | 20.0 | 17.8 |
| Patient 8 | 21.2 | 17.3 | 14.5 | 12.6 |
